# Tailoring of Multisource Deposition Conditions towards Required Chemical Composition of Thin Films

**DOI:** 10.3390/nano12111830

**Published:** 2022-05-27

**Authors:** Jan Gutwirth, Magdaléna Kotrla, Tomáš Halenkovič, Virginie Nazabal, Petr Němec

**Affiliations:** 1 Department of Graphic Arts and Photophysics, Faculty of Chemical Technology, University of Pardubice, Studentská 573, 532 10 Pardubice, Czech Republic; magdalena.kotrla@student.upce.cz (M.K.); tomas.halenkovic@upce.cz (T.H.); 2 CNRS, ISCR-UMR 6226, Université de Rennes 1, F-35000 Rennes, France; virginie.nazabal@univ-rennes1.fr

**Keywords:** thin film, deposition, sputtering, co-sputtering, Ga–Sb–Te, calculation, model

## Abstract

The model to tailor the required chemical composition of thin films fabricated via multisource deposition, exploiting basic physicochemical constants of source materials, is developed. The model is experimentally verified for the two-source depositions of chalcogenide thin films from Ga–Sb–Te system (tie-lines GaSb–GaTe and GaSb–Te). The thin films are deposited by radiofrequency magnetron sputtering using GaSb, GaTe, and Te targets. Prepared thin films are characterized by means of energy dispersive X-ray analysis coupled with a scanning electron microscope to determine the chemical composition and by variable angle spectroscopic ellipsometry to establish film thickness. Good agreement between results of calculations and experimentally determined compositions of the co-deposited thin films is achieved for both the above-mentioned tie-lines. Moreover, in spite of all the applied simplifications, the proposed model is robust to be generally used for studies where the influence of thin film composition on their properties is investigated.

## 1. Introduction

The application potential of many types of materials/thin films is generally determined by some particular property or by a combination of several properties. Basically, these properties intrinsically arise from the material itself and are largely influenced by (i) the qualitative and the quantitative chemical composition, (ii) the short-range order (i.e., chemical (bonding) structure), and (iii) the long-range order (i.e., crystallinity) [1,2,3]. Moreover, these parameters are strongly intertwined. As a result, composition is often used as a default experimental variable because of its easiness to be clearly qualitatively and quantitatively expressed contrary to the other above-mentioned basic matter properties. Moreover, the thin film composition can be directly influenced by the applied deposition conditions in case of multisource depositions [4,5].

Such composition-dependent property studies are the subject of interest in the case of amorphous materials, where, in contrast with crystalline materials, it is possible to obtain (meta)stable homogeneous isotropic material within the broad compositional range called the glass forming region [6,7,8]. Mentioned metastability of amorphous materials generally arises from their thermodynamics when the internal energy of the amorphous state (understood as an undercooled liquid) is higher than the internal energy of the crystalline analogue. Practically, depending on the particular system, the term “metastable” may be assumed to be equal to term “stable”; when under ambient conditions, it means a stable matter for a period of hundreds of years or more—e.g., natural forms of amorphous SiO_2_ (obsidian, moldavite) or artificial medieval silicate glasses. Moreover, and again in contrast with crystalline materials, mathematic functions of physicochemical properties of amorphous materials within the glass forming region typically exhibit continuous behavior, whether monotonous or non-monotonous [6,8,9]. This feature allows fine-tuning of selected property or properties combination.

When considering physical vapor deposition, basically three approaches towards composition-dependent property studies exist. The first approach consists in single-source depositions of the required materials [5,10,11]. This approach, however, requires a set of source materials to cover the entire selected thin film compositional range and, therefore, it is extremely source demanding in term of the materials. Moreover, this method is inapplicable, e.g., for thermal evaporation, where large composition deviations often appear during deposition [12]. The second approach, often called the combinatorial approach or combinatorial deposition [13,14], utilizes typically non-rotating substrates and multiple material sources equipped with deflecting shields or focused on different focal points. The result of such deposition is a substrate covered by a film of variable chemical composition determined by the chosen geometry and deposition conditions. The main limitation of this approach is given by a limited area where the composition of thin film can be assumed as a constant. Thus, such approach is suitable when microscale characterization of the composition and selected properties are available. The last approach, usually called co-deposition, utilizes rotating substrates and multiple material sources in fixed (typically confocal) geometry [4,5,15,16,17]. This experimental setup allows obtaining large-area films with constant composition determined by applied deposition parameters.

Nonetheless, a predictive approach is rarely proposed for the determination of variable co-deposition parameters depending on the required chemical composition. These variables are often estimated *a posteriori* based on the experience and/or determined by feedback from experimental trials [4,5,15,16,17]. It is obvious, that this methodology is time consuming although experimental design approaches are time saving [18]. This is a major drawback of the co-deposition approach, especially in cases of more than two-sources depositions or when high composition accuracy is required.

For the above-mentioned reasons, a simplified model allowing to calculate deposition parameters utilizing basic physicochemical constants of given source materials together with a low number of trial depositions covering basic instrument characteristics (tool-constants) was developed in this work for the case of radiofrequency (RF) magnetron co-sputtering. As a model case, the Ga-Sb-Te ternary system was selected. The choice of this ternary system was made on the basis of particular properties of its thin films, such as large values of optical and electrical contrast originating in crystallization of amorphous, as-deposited thin films. The reported optical changes induced by crystallization of amorphous Ga-Sb-Te amorphous thin films are comparable or even higher than those of prototypical phase-change memory materials based on Ge-Sb-Te ternary [19]. Therefore, Ga-Sb-Te thin films seem to be interesting for potential applications in this field and it is worth studying their fabrication and properties.

## 2. Materials and Methods

Thin films were deposited by conventional RF (13.56 MHz) magnetron (co-)sputtering in Ar plasma [19]. Multi-chamber system MPE600 (Plassys-Bestek, Marolles-en-Hurepoix, France) equipped with symmetrically arranged confocal deposition cluster consisting of three Torus 2 (Kurt J. Lesker, Jefferson Hills, PA, USA) cathodes were used for that purpose. The system was pumped by scroll and turbomolecular pumps to maintain an oil vapor-free vacuum. Plasma was generated by R301 power sources driven by MC2 (both Seren, Vineland, NJ, USA) matchboxes. Process pressure was maintained by feedback loop while being monitored by capacitance gauge.

Pre-cleaned BK7 glass (Crystran, Dorset, UK) and Si wafers (OnSemi, Rožnov pod Radhoštěm, Czech Republic) were used as substrates. Prior to depositions, the surface of the substrates was cleaned in load lock applying RF (13.56 MHz) Ar plasma.

The deposition conditions were as follows: background pressure ≤ 1×10^−5^ Pa (typically ≤ 5×10^−6^ Pa), average target-to-substrate distance of 9 cm, Ar pressure of 0.5 Pa, Ar flow rate of 75 sscm, and substrate holder rotation of 5 rpm. The only variable parameters were the power applied on individual cathodes and deposition duration, as summarized in Table 1.

For the substrate plasma cleaning as well as for the deposition, Ar gas of 99.9999% purity (Linde, Prague, Czech Republic) was used. Two-inch GaSb and GaTe (both 99.999% purity) and Te (99.99% purity, all ALB Materials, Henderson, NV, USA) deposition targets were used.

The thickness of the thin films deposited on BK7 substrates was determined by variable angle spectroscopic ellipsometry (VASE). Due to relatively low band gap energies of studied materials, measurements were carried out from UV to IR region utilizing both UV-Vis-NIR and IR ellipsometers to reach maximum accuracy of the results. Rotating analyser ellipsometer for spectral range of 300–2300 nm and rotating compensator ellipsometer IR-VASE (both J. A. Woollam, Lincoln, NE, USA) for spectral range of 1.7–10 µm were employed. Measurements were performed under three angles of incidence (50°, 60°, and 70°) while a step of 20 nm for UV-Vis-NIR and 8 cm^−1^ for NIR-MIR range were used.

The measured data were modeled with a four-layer model of the sample structure consisting of (i) glass substrate, (ii) thin film, (iii) surface roughness, and (iv) air as ambient. Bruggeman effective medium approximation [20] was utilized for modeling of surface roughness. The measured data of GaSb and Te thin films were modeled using the Cody–Lorentz oscillator model [21]. In the case of GaTe thin films, the model exploiting Kramers–Kronig consistent parametric semiconductor oscillator function [22] was preferably employed.

Elemental analysis of thin films was performed by means of energy dispersive X-ray analysis coupled with the scanning electron microscope (SEM-EDX) technique. IT 300 LA EDS (Jeol, Tokyo, Japan) EDX equipped SEM was employed when primary e-beam accelerating voltage (10 kV) was applied. Thin films deposited on Si substrates were used for the analysis to avoid possible X-ray emission line interferences. Ga-L, Sb-L, and Te-L X-ray emission lines were exploited for the determination of chemical composition. Standardless quantitative analysis based on ZAF Method was applied for EDX spectra evaluation when an uncertainty of less than 2 at% was expected when the atomic ratio of (co-)sputtered thin films was determined.

The structure of the deposited thin films was determined by X-ray diffraction technique. For the measurements in the range of 5–90° with 0.02° step, X-ray diffractometer MiniFlex 600 (Rigaku, Tokyo, Japan) equipped with Cu Kα source was utilized.

## 3. Results

### 3.1. Model

The principle of the developed simplified model is based on the approximation of independent particle fluxes from given sources. Indeed, resulting multisource deposition can be treated as a sum of exploited single source depositions. Then the developed model can be schematically described step-by-step as follows:

1. Test depositions for a single target for fixed Ar pressure and flow within expected (or standardly used) power range to allow determination of device constants for a given experimental setup
2. Determination of the thin film thickness for each power applied to a specific target (cathode) and calculation of the deposition rate
3. Establishment of deposition rate vs. power dependencies and their fitting by suitable functions
4. Determination of the co-deposited thin films composition
5. Calculation of the dependence of material flux vs. applied power
6. Determination of a set of possible combinations of deposition conditions with respect to the required compositions of the thin film (alternatively also resulting in feedback extension of single-source trial depositions power range, if necessary (i.e., step 1.)
7. Selection of suitable deposition conditions from the set of possible deposition conditions with respect to the requirements other than the thin film compositions (thickness, roughness, morphology, etc.).
8. Determination of co-deposition rate and establishment of co-deposition duration with respect to required thin film thickness
9. Verification of the experimental composition and thickness of the co-deposited thin films

### 3.2. Results of Model

In the present study, the RF magnetron sputtering technique [12,23] was chosen. The main advantage of this technique is good reproducibility (especially in comparison with the thermal evaporation technique) and the availability of multisource deposition systems (contrary to e-beam evaporation [12]).

**Figure 1:**
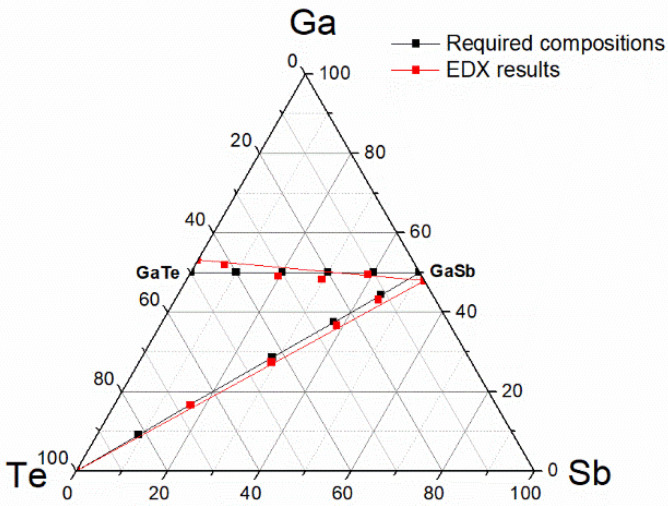
Ternary diagram with compositions of (co-)deposited thin films: as required (black marks), as determined by SEM-EDX (red marks). Corresponding tie-lines connecting uttermost compositions are included as follows: black tie-lines for target nominal compositions, red tie-lines for single source deposited thin films compositions as revealed by SEM-EDX (power of 15 W for GaSb or GaTe and 10 W for Te).

The required composition of the co-sputtered thin films, co-deposited from (i) GaSb and GaTe or (ii) GaSb and Te targets, was defined as A_x_B_100-x_ in pseudobinary expression with an x-step of 20 (GaSb-GaTe and GaSb-Te pseudobinaries, as can be seen in Table 1 and Table 2 and Figure 1), while the required thickness for co-sputtered films was defined as 150 nm.

**Figure 2:**
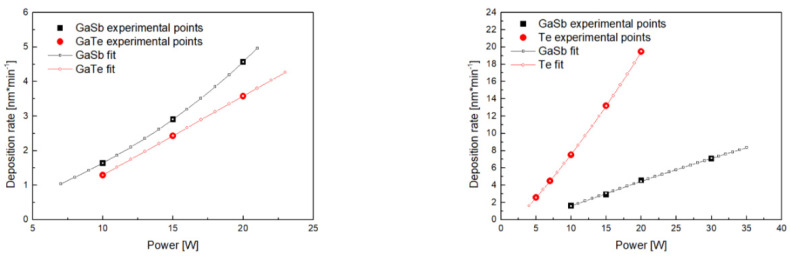
Deposition rate vs. RF power dependencies resulting from experiments (full marks) and as obtained by fitting (empty marks/line) within the range used for co-depositions: GaSb-GaTe (**left**) and GaSb-Te (**right**) pseudobinaries.

Basically, three depositions differing in applied power (10 W, 15 W, and 20 W) were performed per source target during the initial trial depositions. This power range usually guarantees an acceptably high deposition rate while preserving targets from possible damage. The initial pre-deposition calculation was performed on basis of these data. Such an initial pre-deposition calculation serves for determination of expected requisite trial depositions power range covering the requested compositional range. Extension of the power range for GaSb and Te sources was found to be suitable to increase experiment accuracy. Finally, 12 trial depositions were performed (GaSb—4 depositions, GaTe—3 depositions, and Te—5 depositions) to cover the expected power range necessary for all the final co-depositions (Table 1, Figure 2).

The thickness of thin films was determined by VASE. The good agreement between experimentally obtained ellipsometry data and the model fits is exemplified in Supplementary data (Figures S2–S5). The resulting thicknesses of thin films and calculated deposition rates are summarized in Table 1 and Figure 2.

The obtained deposition rate vs. power dependencies were then fitted by the appropriate function. However, this function is generally unknown: linear growth [24,25,26], monotonic degressive [27,28], or progressive [29] growth, or complex non-monotonical course [30] were reported. In order to obtain precise results based on available data, exponential function (Equation (1)) in the range of 10–15–20 W was used for GaSb. Further GaSb trial depositions power extension to 10–15–20–30 W range led to the redefinition of GaSb fitting function to linear one (Equation (2)). Linear function in the range of 10–15–20 W was employed for GaTe in the case of GaSb-GaTe co-depositions. Exponential function in range 5–7–10–15–20 W was utilized for Te in case of GaSb-Te co-depositions.

(1) $$DR_{ssd}=c_{1}\cdot e^{\frac{P}{c_{2}}}+c_{3}$$

(2) $$DR_{ssd}=c_{4}\cdot P+c_{5}$$

where *DR_ssd_* is deposition rate for single source deposition [nm·min^−1^], *c*_1_, *c*_2_, *c*_3_, *c*_4_, *c*_5_ are tool constants for a given setup, and *P* is RF power [W].

The used functions serve only as mathematical formulae providing precise fits within a given region without any specific/intrinsic physical meaning of equations parameters. Determined deposition rates of trial depositions as well as the fit are presented in Figure 2 and Supplementary data (Tables S2 and S3).

The chemical composition of deposited thin films was determined by SEM-EDX technique and is summarized in Table 2.

As established by X-ray diffraction results, the GaSb and GaTe thin films were found to be amorphous, while the Te thin films were found to be at least partially crystalline because diffractions of hexagonal tellurium (PDF card no: 00-004-0554) appeared in the measured diffractograms.

It is crucial to express deposition rate as a molar flux for stoichiometry calculations. This can be done easily—when constant unitary deposition area (e.g., 1 m^2^) is assumed, then the molar flux can be expressed as Equation (3)

(3) $$MF=\frac{\frac{10^{-9}\cdot d}{t}\cdot \rho }{M}$$

where *MF* is molar flux [mol·min^−1^·m^−2^], *d* is film thickness [nm], *t* is deposition duration [min], *ρ* is density of material [kg·m^−3^], and *M* stands for molar mass [kg·mol^−1^].

Fundamentally, such expression of molar fluxes allows finding their possible combinations (and hence possible combinations of deposition powers) fulfilling requested composition ratios. Usually, more than one possible combination of deposition powers occurs for any particular thin film composition with acceptable accuracy. Therefore, additional parameter/s (as, e.g., the smallest difference in power between calculation result and hardware option (typically determined by power setting scaling spacing) or similar overall deposition rate of all the co-depositions) can be considered for final selection. In this study, both mentioned factors were taken into account. Allowed power setting scaling spacing of 1 W was available, and thus, the maximum deviation in power between calculation result and hardware option is 0.5 W. Average deposition rate of ~5–6 and ~10–12 nm·min^−1^ was achieved for most of the prepared thin films of GaSb-GaTe and GaSb-Te pseudo-binaries, respectively. The aspect of accuracy was highlighted in the presented study. This fact, together with the high slope of power dependency of the deposition rate of tellurium, lead to a broader range of deposition rates in the case of GaSb-Te co-depositions than in the case of GaSb-GaTe co-depositions. Nevertheless, an extraordinary elevation of deposition rate can be seen only in the case of the tellurium richest sample from the GaSb-Te tie-line (16.92 nm·min^−1^).

Finally, deposition duration *t* with respect to required co-deposited thin film thickness was established. For simplification, the necessary overall co-sputtering deposition rate was considered as a sum of calculated deposition rates of corresponding single source depositions (SSD), which is expressed in Equation (4).

(4) $$t=\frac{d}{DR_{cd}}=\frac{d}{\sum_{i=1}^{n}{\left(DR_{ssd}\right)}_{i}}$$

where *d* is co-deposited thin film thickness [nm] and *DR_cd_* is co-deposition rate [nm·min^−1^].

## 4. Discussion

The aim of this work is to compile an approach having a low requirement of input data, high versatility, and high accuracy. Therefore, several simplifications (some of them determinable as physically incorrect from a rigorous viewpoint) were introduced.

Externally required input parameters of the model are only atomic masses and materials densities, while other parameters—deposition power and corresponding thin film thickness/composition—result from the experiments.

While atomic masses are well known and widely published, and molar masses can be calculated by standard formula, density data availability is more problematic. Generally, as highlighted in the present study, two problems (may) arise—preparation of unusual compositions and/or amorphous nature of the material. Both these factors often lead to the unavailability of the correct density data. Moreover, this problem plays a role in both input data (i.e., the density of thin films obtained from single source trial depositions) as well as output data (i.e., the density of thin films obtained from co-deposition). The first mentioned issue consists in the inaccuracy of single-source molar flux determination and results in the inaccuracy of composition of co-deposited thin films. The second mentioned issue consists in the inaccuracy of calculation of deposition rate of co-deposited thin films and results in the inaccuracy of final co-deposition duration determination and hence final co-deposited thin films thicknesses.

In the frame of this study, densities of crystalline GaSb, GaTe, and Te (Supplementary data, Table S1) were used, while no correction to the amorphous state of GaSb and GaTe thin films was done. However, following glass forming theory [8,31,32,33], the density of GaSb and GaTe could be generally reduced by several per cent to obtain higher accuracy of the model. Densities of resulting amorphous Ga-Sb-Te thin films can be classified as generally unknown. For that reason, the resulting thicknesses of co-deposited thin films were simply assumed/calculated as the sum of thicknesses of equivalent single source deposited thin films. This is a rough simplification when handling thickness and hence volume as additive physical quantity instead of mass, because of volume expansion or contraction effects. However, there is no possibility of a simple approximation of density. Sometimes, this can be evaded by the utilization of appropriately (i.e., mass) weighted linear combination of densities of individual components (i.e., densities of single source deposited thin films). On the other hand, this approach may be contradictory when new phases occur. Such a phenomenon is quite common, especially when broad-range composition series are prepared. This issue becomes highlighted in the presented study when densities of constituting elements, i.e., *ρ_Ga_* = 5.90 g·cm^−3^, *ρ_Sb_* = 6.69 g·cm^−3^, and *ρ_Te_* = 6.24 g·cm^−3^ are compared with densities of compounds used for deposition—i.e., *ρ_GaSb_* = 5.61 g·cm^−3^, and *ρ_GaTe_* = 5.44 g·cm^−3^ [34,35]. It is clearly visible that volume expansion takes place and compound densities are lower than the densities of all the constituent elements. Indeed, the above-described simplification was used for the highest possible clarity at an expected price of lower accuracy of the calculation or, more exactly written, calculation-based estimations. Better accuracy can be achieved while precise density data will be used. There are several possibilities to determine the density of thin films experimentally, for example, non-destructively in situ via X-ray reflectivity measurements [36] or destructively ex-situ by any quantitative chemical analysis [37].

It is necessary to mention that some inherent instability of the deposition rate up to ±5% was detected. This effect can be observed when deposition rates of the single source trial depositions and the single source final depositions are compared. The origin of depicted instability, which subsequently results in the inaccuracy of the deposition rate determination and further inaccuracy of composition of co-sputtered thin films, can be at least partially ascribed to an automatic matching mode used by the deposition system. Here, optimal conditions of glow discharge are guaranteed by capacitance changes within the matching unit; this process is connected with the deposition rate change. It is worthy to note that the absolute value of mentioned inaccuracy is smaller than deposition rate change, which is connected with the power scaling spacing of the RF-power source (i.e., 1 W), as can be seen in Table 1, Tables S2, and S3 and Figure 2.

The impact of such inaccuracy (which is also determined by stoichiometric ratios) on the composition of co-deposited thin films is similar to that of SEM-EDX technique accuracy. Moreover, this inaccuracy of deposition rates is effectively reduced for the purpose of multisource depositions by fitting deposition rate-power dependency by a given mathematic function.

Because different shapes of deposition rate vs. power dependency were previously reported [23,24,25,26,27,28,29], linear or exponential functions based on currently known trial depositions values were applied in order to obtain precise fits as described in the Results Section. As shown in Figure S2, the originally proposed exponential function for GaSb within the 10–15–20 W power range was proven to be inappropriate when 30 W point, which was necessary to be added for GaSb-Te co-depositions, was considered.

It is obvious that the fitting functions do not intercept [0, 0] point (i.e., zero deposition rate for zero power applied on the cathode). This is not contradictory because as written above, the fitting functions were used only for a given power range. Depending on applied power, glow discharge can be categorized by particle interactions/energies into several regimes (subthreshold regime, knock-on regime, linear cascade regime, implantation regime) [17,38], while glow discharge physics within these regimes differ. Consequently, the universal formula covering the whole power range will be more complex containing more terms. However, some of the terms can be neglected within the power range covered in this study and hence cause observed discrepancy for [0, 0] point.

As one can deduct from the results, used functions describe dependencies correctly for both interpolated and extrapolated points (Figure 2). Nonetheless, increased content of GaTe (or more exactly of Ga because of non-stoichiometry of single source GaTe deposited thin film) for compositions of uttermost GaSb-GaTe co-deposited thin films can be at least partially counted on the improper fitting function of GaSb, causing overestimation of GaSb deposition rate (Supplementary data, Figure S1). It should be mentioned that among power ranges covered in this study, the most experimentally exposed power values are the low power extrapolated points where problems with plasma ignition or even plasma stability may arise. Moreover, particularly in the case of tellurium, due to low absolute value and high slope of dependency (Figure 2), the impact of possible power inaccuracy or fluctuations will be more significant than in other cases. Nevertheless, the results (Table 1 and Table 2) do not show any elevated level of inaccuracy.

Some differences between calculated/estimated and measured co-deposited thin film thicknesses were observed. These differences are up to circa 20 and 10% for GaSb-GaTe and GaSb-Te co-depositions, respectively. As mentioned above, this discrepancy can be connected with used simplification regarding materials’ densities.

A change of thin film stoichiometry with respect to the corresponding target was observed in the case of GaSb and GaTe, even within single-source depositions. For the power of 15 W, GaSb thin films are slightly depleted in Ga (Ga_48.0_Sb_52.0_) while GaTe thin films are, to a small extent, Ga enriched (Ga_53.1_Te_46.9_), as depicted in Table 2 and Figure 1. Moreover, a certain trend of composition vs. power dependency can be observed; applied power increase causes a mild decrease of the composition deviation for GaSb as well as for GaTe thin films within the applied power range.

The following part will be focused on compound–compound (i.e., GaSb-GaTe) co-depositions. The effects of both the above-mentioned uncertainties on composition are close to the accuracy of SEM-EDX technique and hence neglected in the frame of the presented calculations (hereafter case A).

While the standalone composition deviations between targets and corresponding thin films during single source depositions (hereafter case B) can be generally introduced into the calculation relatively easily, the power dependency of these deviations (hereafter case C) leads in a broader scale viewpoint to a significant change of calculation.

As mentioned above, the aim of the presented study is to predict the composition of the co-deposited thin film just as a function of power applied to individual cathodes and sputtering targets` nominal composition (case A, Equations (5)–(8), Table 3).

The introduction of power-independent stoichiometry deviations (case B) causes the only change of nominal target compositions to corresponding compositions of single source deposited films, including changes of molar masses and densities, i.e., the co-deposited thin film composition becomes a function of applied powers and single source deposited thin films composition (case B, Equations (5)–(8), Table 3). Again, the most difficult is a treatment of density where, due to the high probability of non-stoichiometric composition of thin films and thus, high probability of density data unavailability, some density approximation will probably be necessary.

If power-dependent compositional deviations are considered (case C), the co-deposited thin film composition becomes a function of applied powers and corresponding individual single source deposited thin films compositions, including changes of molar masses and densities, i.e., new variables will be involved (Equations (5)–(8), Table 3). Due to time/material costs, this means the necessity of expression of composition deviation as a function of applied power as well, including calculation of corresponding molar weights and corresponding densities for each possibly applied power. Moreover, while previous simplified calculation (cases A and B) preserves ratios of chemical elements of particular sources constant, fluctuations of these ratios become allowed in case C. This, in fact, consequently, leads to the separation of the overall chemical balance of source compounds towards the chemical balance of particular elements. This newly arising chemical balance of individual elements is essentially interrelated through target material stoichiometry but shifted by particular deviations. These new variables also cause the coupling of suitable deposition conditions becomes (contrary to cases A and B) a matter of multidimensional input data (applied powers and corresponding single source deposited thin film compositions) and hence the calculation will be more complicated, especially for manual handling.

The next consequence of described facts is that the calculation results of thin film compositions in case A follow tie-line between nominal compositions of targets (Figure 2, black lines). In case B, the calculation results follow the tie-line between single source deposited thin film compositions (Figure 2, red lines). In case C, the calculation results will follow some hypothetical deviation determined tie-curve between single source deposited thin film compositions. Or, even more exactly, when reflecting the existence of more than one suitable combination of powers applied on individual cathodes, calculation results may cover composition deviation determined tie-area.

For the compound-compound (GaSb-GaTe) co-deposition (Equation (5)), the balances for involved elements (i.e., Ga (Equation (6)), Sb (Equation (7)), and Te (Equation (8))) can be described for all the above mentioned cases (A–C) together with clarification (Table 3) of all the parameters and their type (constant, variable, etc.) as follows:

(5) $$\alpha \;{Ga}_{A}{Sb}_{B}\;+\;\beta \;{Ga}_{C}{Te}_{D}\;\to \;\gamma \;{Ga}_{X}{Sb}_{Y}{Te}_{Z}$$

(6) γ. X =α. (A+ ΔA)+β. (C+ ΔC)

(7) γ. Y = α. (B+ ΔB)=α. (B− ΔA)=α. (1−A− ΔA)

(8) γ. Z =β. (D+ ΔD)=β. (D− ΔC)=β. (1−C− ΔC) 

where *α*, *β*, *γ* are molar deposition fluxes, *A*, *B*, *C*, *D*, *X*, *Y*, *Z* are stoichiometric coefficients, *ΔA*, *ΔB*, *ΔC*, *ΔD* are composition deviations between the single source deposited thin film and the corresponding target.

It should be mentioned, that parameter *γ* was added just for maximum description accuracy. This parameter is interconnected with parameters α and *β* through the stoichiometric coefficients of the compounds (Equation (5)). However, under given assumptions and in the frame of the thin film ”synthesis” chemical reaction description, the *γ* parameter is allowed to be assumed constant and equal to 1, while *α* and *β* become equal to stoichiometric ratios.

While case A calculation requires only two variables reflecting power dependency of deposition rate for each employed cathode and four (reducible to two) constants reflecting stoichiometry of particular targets, case B calculation requires another four (reducible to two) constants reflecting composition deviation between single source deposited thin film and corresponding target. In comparison with case A calculation, case C requires another four (reducible to two) variables reflecting the power dependency of the composition deviation between the thin film and corresponding target.

Introduction of stoichiometry change between target and corresponding single source deposited thin film (case B) seems to have a capacity to improve calculation accuracy without any special requirements or other inaccuracy sources (except density approximation as mentioned above). The only additional condition is the knowledge of the single source deposited thin film composition prior to co-deposition experiments. Contrary to that, the introduction of power dependency of composition deviation between targets and corresponding single source deposited thin films drastically changes the method of calculation and significantly increases its difficulty, especially when the coupling is performed manually. Moreover, further variables are introduced, which may negatively influence calculation accuracy. Hence while the benefit is questionable, possible disadvantages are clearly identified.

Unfortunately, because the composition of single source deposited thin films was not known prior to co-depositions, mentioned improvements (case B or C) cannot be verified due to the differences in input data (nominal compositions used for presented thin film depositions and real compositions of single source deposited thin films utilized in case B or C). Only some qualitative comparison may be done on the basis of current data. Under the assumption of constant densities, when compared, molar masses for nominal and real composition of single source deposited thin films (Table 4), a negligible difference (~1%) in the case of GaSb and a moderate difference (~−5%) in case of GaTe appeared. This can be interpreted as that the real molar flux of Ga-Sb is slightly underweighted and the real molar flux of Ga-Te is moderately overweighted in comparison to nominal compositions used for calculations. This is in good agreement with obtained data, when the compositions of all the GaSb-GaTe co-deposited thin films are shifted towards GaTe (Figure 1).

Finally, to further improve the calculation accuracy, the difference between calculated power and power allowed due to setting scaling spacing can be considered. Contrary to previous ones, this approach is reversed when no calculated but required composition is refined. The bottom line of this refinement results from the difference between calculated power and power allowed due to setting scaling spacing and from absolute value and slope of molar flux power dependence. As written in the Results Section, the effect of this improvement should generally be close to or below of accuracy of the SEM-EDX technique. In the case of the present study, differences are in tenths of atomic percent (maximum difference is circa 0.5 at%), i.e., far below the difference between nominal target compositions and single source deposited thin film compositions.

## 5. Conclusions

A methodology for the selection of multi-target deposition conditions to obtain thin films of the required chemical composition has been successfully developed in the case of magnetron RF co-sputtering. A simplified physico–chemical calculation approach based on single target experiments has been introduced, assuming that the deposition sources do not interact significantly, impacting the sputtering rate of single targets. The aforementioned experiments were performed in the ternary Ga-Sb-Te system, specifically on the GaSb-GaTe and GaSb-Te pseudobinary tie-lines using the single GaSb, GaTe, and Te targets.

An important point of this study is the possibility of considering the deviations of the chemical composition of the thin films obtained with respect to the initial stoichiometry of the single targets and also to be able to compensate for the deviations in the deposition rates of these single sources. Indeed, a deviation in stoichiometry up to ~2 and ~3 at% for GaSb and GaTe, respectively, was observed for single source depositions. Inaccuracy typically up to ±5% has been found for the deposition rate of the single source depositions. Nevertheless, the influence of such a deposition rate inaccuracy on the composition of the co-deposited thin films is effectively reduced by using appropriate fitting functions.

Good agreement between the calculated results of the presented model and the experimentally determined compositions of the co-sputtered thin films was achieved for both tie-lines mentioned above. When the compositional differences between source compounds and corresponding single source deposited thin film is not considered, the inaccuracy of the composition is up to ~3.5 and ~1.5 at% for GaSb-GaTe and GaSb-Te pseudobinaries, respectively. Thus, the inaccuracy in absolute values is practically equal to composition deviations observed for source compounds (i.e., GaSb and GaTe targets) and the corresponding thin films obtained by its single source depositions.

With the considered simplifications, an acceptable agreement between the results of calculation-based estimation and experimentally determined deposition rates of co-deposited thin films was achieved for both the above-mentioned tie-lines. The deposition rate inaccuracy was found to be up to ~20 and ~10% for GaSb-GaTe and GaSb-Te co-depositions tie-lines, respectively.

Despite the simplifications, the proposed model is found to be sufficiently robust to provide suitable data applicable for co-depositions of multi-component thin films to predict the chemical compositions of RF sputtered films based on the information from the sputtering of individual targets.

## Figures and Tables

**Table 1:** Deposition conditions, thicknesses of prepared thin films, and deposition rates.

Required Composition			Deposition Conditions				Thickness		Deposition Rate		
			Power			Duration	Measured	Estimated	Measured	Estimated	Difference
GaSb	GaTe	Te	Cat1	Cat2	Cat3						Meas-Est
[mol%]	[mol%]	[at%]	[W]	[W]	[W]	[min:s]	[nm]	[nm]	[nm·min^−1^]	[nm·min^−1^]	[%]
100	-	-	10	-	-	60	98	-	1.64	-	-
100	-	-	15	-	-	60	174	-	2.90	-	-
100	-	-	20	-	-	60	274	-	4.57	-	-
100	-	-	30	-	-	30	212	-	7.08	-	-
-	100	-	-	10	-	60	77	-	1.29	-	-
-	100	-	-	15	-	60	145	-	2.42	-	-
-	100	-	-	20	-	60	215	-	3.58	-	-
-	-	100	-	-	5	60	154	-	2.56	-	-
-	-	100	-	-	7	30	135	-	4.50	-	-
-	-	100	-	-	10	60	451	-	7.51	-	-
-	-	100	-	-	15	60	793	-	13.21	-	-
-	-	100	-	-	20	60	1167	-	19.46	-	-
100	0	-	15	0	-	60:00	168	150	2.81	2.90 T	−3.28
80	20	-	21	10	-	23:45	146	150	6.16	6.25	−2.31
60	40	-	16	14	-	27:45	170	150	6.14	5.40	13.71
40	60	-	12	18	-	28:45	183	150	6.37	5.22	21.94
20	80	-	7	23	-	27:15	166	150	6.08	5.30	14.45
0	100	-	0	15	-	60:00	158	150	2.63	2.42 T	8.68
80	-	20	35	-	4	15:0	156	150	10.42	9.96	4.62
60	-	40	30	-	6	14:15	148	150	10.35	10.61	−2.17
40	-	60	25	-	9	12:15	149	150	12.18	12.32	−0.82
20	-	80	20	-	15	8:30	144	150	16.92	17.63	−3.77
0	-	100	-	-	10	20:00	141	150	7.06	7.51 T	−6.06

Deposition conditions (power [W] and deposition duration [min:s]), film thickness [nm] determined by VASE (±2 nm), and calculated deposition rate [nm·min^−1^] of trial depositions. Calculated deposition conditions (power [W], deposition duration [min:s]) of co-sputtered thin films together with calculated deposition rate [nm·min^−1^], film thickness [nm] determined by VASE (±2 nm), and deposition rate [nm·min^−1^] calculated on the basis of VASE results. The difference between the deposition rate calculated and obtained from VASE results are related to the calculated value. Cat1, Cat2, and Cat3 stand for cathodes 1, 2, and 3. T letter marks comparison between trial and final single source deposition. Yellow backgrounded fields are inappropriate in the case of a particular deposition.

**Table 2:** Required and experimentally determined thin film composition.

Required Composition						Real Composition			Difference (Real-Required)		
Pseudobinary Expression			Atomic Expression			Atomic Composition			Atomic Composition		
GaSb	GaTe	Te	Ga	Sb	Te	Ga	Sb	Te	Ga	Sb	Te
[mol%]	[mol%]	[at%]	[at%]	[at%]	[at%]	[at%]	[at%]	[at%]	[at%]	[at%]	[at%]
100	0	-	50.0	50.0	0.0	48.0	52.0	0.0	−2.0	2.0	0.0
80	20	-	50.0	40.0	10.0	49.5	39.0	11.5	−0.5	−1.0	1.5
60	40	-	50.0	30.0	20.0	48.2	29.6	22.1	−1.8	−0.4	2.1
40	60	-	50.0	20.0	30.0	49.0	19.6	31.4	−1.0	−0.4	1.4
20	80	-	50.0	10.0	40.0	51.9	6.5	41.6	1.9	−3.5	1.6
0	100	-	50.0	0.0	50.0	53.1	0.0	46.9	3.1	0.0	−3.1
80	-	20	44.4	44.4	11.1	43.1	44.5	12.4	−1.3	0.0	1.3
60	-	40	37.5	37.5	25.0	36.7	38.7	24.7	−0.8	1.2	−0.3
40	-	60	28.6	28.6	42.9	27.4	29.0	43.6	−1.2	0.4	0.7
20	-	80	16.7	16.7	66.7	16.6	16.8	66.7	−0.1	0.1	0.0
0	-	100	0.0	0.0	100.0	0.0	0.0	100.0	0.0	0.0	0.0

Required composition [atomic/molar %] expressed in pseudobinary form and atomic form and composition of deposited thin films as revealed by SEM-EDX (±2 at%). Enumeration of compositional difference between SEM-EDX determined composition of (co-)deposited thin film and required composition of (co-)deposited thin film for both studied tie-lines (i.e., GaSb-GaTe, and GaSb-Te).

**Table 3:** Parameters of chemical balance description for cases A, B, and C. Variable (f(P)) means variable which is a function of applied power.

Parameter	Case A	Case B	Case C
*α, β*	Variable (f(*P*))	Variable (f(*P*))	Variable (f(*P*))
*γ*	Const (1.0)	Const (1.0)	Const (1.0)
*A, B, C, D*	Const (0.5)	Const (0.5)	Const (0.5)
*ΔA*	Const (0)	Const (*ΔA*)	Variable (f(*P*))
*ΔB*	Const (0)	Const (*ΔB* = −*ΔA*)	Variable (f(*P*))
*ΔC*	Const (0)	Const (*ΔC*)	Variable (f(*P*))
*ΔD*	Const (0)	Const (*ΔD* = −*ΔC*)	Variable (f(*P*))

**Table 4:** Molar masses [34] of nominal compositions and compositions of real single target deposited thin films.

Composition	Ga_50_Sb_50_	Ga_48.0_Sb_52.0_	Ga_50_Te_50_	Ga_53.1_Te_46.9_
**Molar mass [g·mol^−1^]**	191.48	193.60	197.32	188.25

## Data Availability

Not applicable.

## Supplementary Materials

The following supporting information can be downloaded at: https://www.mdpi.com/article/10.3390/nano12111830/s1, Supplementary data.
