# Supplementary material for: Tailoring of Multisource Deposition Conditions towards Required Chemical Composition of Thin Films
Source: Nanomaterials (Basel). 2022 May 27;12(11):1830. doi: 10.3390/nano12111830 (PMC9182166; doi:10.3390/nano12111830)
Supplement: Supplementary file 1 [file nanomaterials-12-01830-s001.zip › nanomaterials-1712797-supplementary.pdf]

nanomaterials

Article

Supplementary Data: Tailoring of Multisource Deposition Conditions towards Required Chemical Composition of Thin Films

Jan Gutwirth [1,*], Magdaléna Kotrla [1], Tomáš Halenkovič [1], Virginie Nazabal [1,2] and Petr Němec [1,*]

[1] Department of Graphic Arts and Photophysics, Faculty of Chemical Technology, University of Pardubice, Studentská 573, 532 10 Pardubice, Czech Republic; magdalena.kotrla@student.upce.cz (M.K.); tomas.halenkovic@upce.cz (T.H.)
[2] CNRS, ISCR-UMR 6226, Université de Rennes 1, F-35000 Rennes, France; virginie.nazabal@univ-rennes1.fr
* Correspondence: jan.gutwirth@upce.cz (J.G.); petr.nemec@upce.cz (P.N.)

1. Supplementary Results

Table S1. Model input parameters.

	GaSb	GaTe	Te
**Density ϱ [kg·m^{-3}]**	5610	5440	6240
**Molar/Atomic mass M [kg·mol^{-1}]**	0.19148	0.19732	0.12760

Table S2. Fits of deposition rates of GaSb-GaTe co-deposition. Black ink represents interpolated points while red ink represents extrapolated points with respect to the performed trial depositions.

Power	GaSb	GaTe	GaSb	GaTe	GaSb	GaTe
[W]	[m·min^{-1}] 1×10^{-9}	[m·min^{-1}] 1×10^{-9}	[kg·min^{-1}] 1×10^{-12}	[kg·min^{-1}] 1×10^{-12}	[mol·min^{-1}] 1×10^{-12}	[mol·min^{-1}] 1×10^{-12}
7	1.04		0.18		0.97	
8	1.23		0.22		1.14	
9	1.43		0.25		1.33	
10	1.64	1.29	0.29	0.24	1.53	1.20
11	1.86	1.52	0.33	0.28	1.74	1.42
12	2.10	1.75	0.37	0.32	1.96	1.63
13	2.35	1.98	0.42	0.36	2.19	1.84
14	2.62	2.21	0.47	0.41	2.44	2.06
15	2.90	2.44	0.52	0.45	2.70	2.27
16	3.20	2.66	0.57	0.49	2.98	2.48
17	3.51	2.89	0.63	0.53	3.27	2.70
18	3.85	3.12	0.69	0.57	3.58	2.91
19	4.20	3.35	0.75	0.62	3.91	3.12
20	4.57	3.58	0.81	0.66	4.25	3.34
21	4.96	3.81	0.88	0.70	4.62	3.55
22		4.04		0.74		3.76
23		4.27		0.78		3.98

Table S3. Fits of deposition rates of GaSb-Te co-deposition. Black ink represents interpolated points while red ink represents extrapolated points with respect to performed trial depositions.

Power	GaSb	Te	GaSb	Te	GaSb	Te
[W]	[m·min^{-1}] 1 × 10^{-9}	[m·min^{-1}] 1 × 10^{-9}	[kg·min^{-1}] 1 × 10^{-12}	[kg·min^{-1}] 1 × 10^{-12}	[mol·min^{-1}] 1 × 10^{-12}	[mol·min^{-1}] 1 × 10^{-12}
4		1.62		0.26		2.03
5		2.55		0.41		3.21
6		3.51		0.56		4.41
7		4.49		0.72		5.63
8		5.49		0.88		6.89
9		6.51		1.04		8.18
10	1.59	7.56	0.28	1.21	1.48	9.49
11	1.88	8.63	0.34	1.38	1.75	10.84
12	2.18	9.73	0.39	1.56	2.03	12.22
13	2.47	10.85	0.44	1.74	2.30	13.63
14	2.76	12.00	0.49	1.92	2.57	15.07
15	3.05	13.17	0.54	2.11	2.84	16.54
16	3.34	14.37	0.59	2.30	3.10	18.05
17	3.62	15.61	0.65	2.50	3.37	19.60
18	3.90	16.86	0.70	2.70	3.63	21.18
19	4.18	18.15	0.74	2.91	3.89	22.80
20	4.46	19.47	0.79	3.12	4.15	24.45
21	4.73		0.84		4.40	
22	5.00		0.89		4.66	
23	5.27		0.94		4.91	
24	5.54		0.99		5.16	
25	5.80		1.03		5.40	
26	6.07		1.08		5.65	
27	6.33		1.13		5.89	
28	6.59		1.17		6.13	
29	6.85		1.22		6.37	
30	7.10		1.27		6.61	
31	7.35		1.31		6.85	
32	7.60		1.36		7.08	
33	7.85		1.40		7.31	
34	8.10		1.44		7.54	
35	8.35		1.49		7.77	

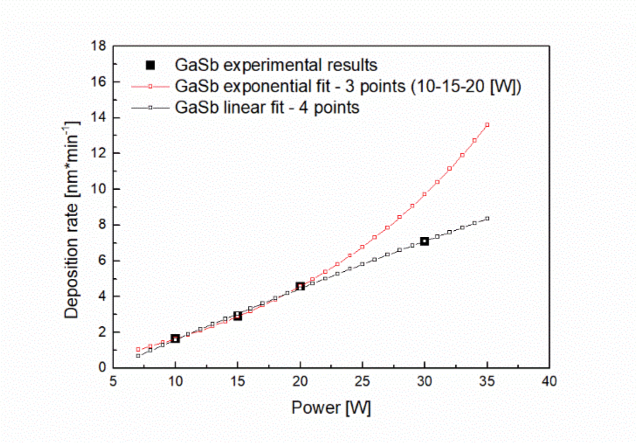

Figure S1. Comparison of deposition rate on deposition power dependency for GaSb. Experimentally obtained points (full marks) and fits - 3-point (10–15–20 W) exponential fit used for GaSb-GaTe co-depositions in range 7–21 W (red empty marks/line) and 4-point (10–15–20–30 W) linear fit used for GaSb-Te co-depositions in range 20–35 W (black empty marks/line).

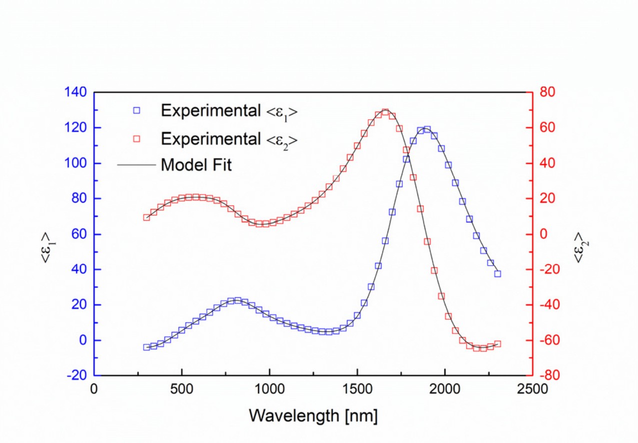

Figure S2. The comparison of experimentally obtained data and the model fit for GaSb thin film sputtered at power of 10 W. The experimental real <ε1> and imaginary <ε2> parts of pseudodielectric function were determined by UV-Vis-NIR ellipsometer (angle of incidence: 70°).

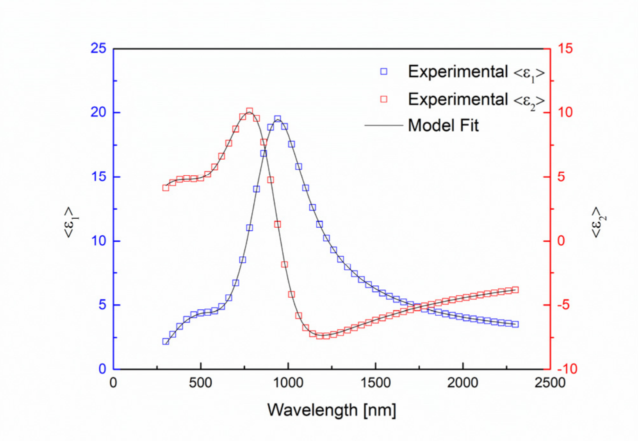

Figure S3. The comparison of experimentally obtained data and the model fit for GaTe thin film sputtered at power of 10 W. The experimental real <ε1> and imaginary <ε2> parts of pseudodielectric function were determined by UV-Vis-NIR ellipsometer (angle of incidence: 70°).

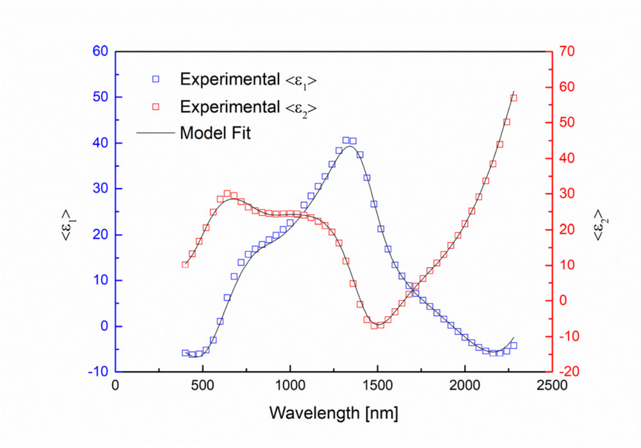

Figure S4. The comparison of experimentally obtained data and the model fit for Te thin film sputtered at power of 10 W. The experimental real <ε1> and imaginary <ε2> parts of pseudodielectric function were determined by UV-Vis-NIR ellipsometer (angle of incidence: 70°).

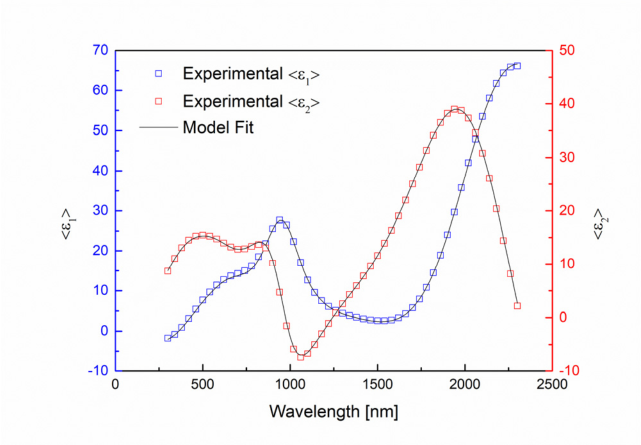

Figure S5. The comparison of experimentally obtained data and the model fit for co-sputtered Ga-Sb-Te thin film (the applied power on cathodes: GaSb 30 W and Te 6 W). The experimental real <ε1> and imaginary <ε2> parts of pseudodielectric function were determined by UV-Vis-NIR ellipsometer (angle of incidence: 70°).
